# Impact of Generic Entry on Hospital Antimicrobial Use: A Retrospective Quasi-Experimental Interrupted Time Series Analysis

**DOI:** 10.3390/antibiotics10101149

**Published:** 2021-09-24

**Authors:** Mercè Espona, Daniel Echeverria-Esnal, Sergi Hernandez, Alexander Almendral, Silvia Gómez-Zorrilla, Enric Limon, Olivia Ferrandez, Santiago Grau

**Affiliations:** 1 Department of Pharmacy, Hospital del Mar, Passeig Maritim 25-29, 08003 Barcelona, Spain; mespona@psmar.cat (M.E.); dechevarria@psmar.cat (D.E.-E.); oferrandez@psmar.cat (O.F.); 2 Infectious Pathology and Antimicrobials Research Group (IPAR), Institut Hospital del Mar d’Investigacions Mèdiques (IMIM), Dr. Aiguader 88, 08003 Barcelona, Spain; sgomezzorrilla@psmar.cat; 3 VINCat Program Surveillance of Healthcare Related Infections in Catalonia, 08097 Barcelona, Spain; shernandezbaeza@iconcologia.net (S.H.); alexanderalmendral@iconcologia.net (A.A.); elimon@iconcologia.net (E.L.); 4 Department of Infectious Diseases, Hospital del Mar, Passeig Maritim 25-29, 08003 Barcelona, Spain; 5 Department of Pharmacology, Universitat Autònoma de Barcelona, 08193 Barcelona, Spain

**Keywords:** antibiotic policy, generic antibiotics, branded antibiotics, antibiotics, defined daily doses, antimicrobial consumption

## Abstract

Background: The impact of antimicrobials generic entry (GE) is controversial. Their introduction could provide an economic benefit yet may also increase their consumption, leading to a higher risk of resistance. Our aim was to analyze the impact of GE on trends of antimicrobial consumption in an acute-care hospital. Methods: A retrospective quasi-experimental interrupted time series analysis was conducted at a 400-bed tertiary hospital in Barcelona, Spain. All antimicrobials for systemic use for which a generic product entered the hospital from January 2000 to December 2019 were included. Antimicrobial consumption was expressed as DDD/100 bed days. Results: After GE, the consumption of cefotaxime (0.09, *p* < 0.001), meropenem (0.54, *p* < 0.001), and piperacillin-tazobactam (0.13, *p* < 0.001) increased, whereas the use of clindamycin (−0.03, *p* < 0.001) and itraconazole (−0.02, *p* = 0.01) was reduced. An alarming rise in cefepime (0.004), daptomycin (1.02), and cloxacillin (0.05) prescriptions was observed, despite not achieving statistical significance. On the contrary, the use of amoxicillin (−0.07), ampicillin (−0.02), cefixime (−0.06), fluconazole (−0.13), imipenem–cilastatin (−0.50) and levofloxacin (−0.35) decreased. These effects were noticed beyond the first year post GE. Conclusions: GE led to an increase in the consumption of broad-spectrum molecules. The potential economic benefit of generic antibiotics could be diluted by an increase in resistance. Antimicrobial stewardship should continue to monitor these molecules despite GE.

## 1. Introduction

The introduction of generic drugs in the pharmaceutical industry necessitates addressing the essential issue of efficiency [1]. In recent years, due to the limitation of economic resources allocated to the acquisition of drugs in the different health systems, the use of generic drugs has increased. In an evolving world where drug-associated costs increase every year, the use of generics is, therefore, essential to maintain budget balance. The development of generic drugs also exhibits other advantages. For example, reducing their price allows them to be available in areas of the world where they would not otherwise be found, saving a considerable number of lives [2].

Economic costs are among the items that antimicrobial stewardship teams need to evaluate and optimize. Unfortunately, the development of the antimicrobial resistance pandemic supposes an important economic investment [3]. The use of novel molecules is normally associated with high prices. Antimicrobial stewardship teams, due to their limited resources, usually focus their attention on these molecules. On the contrary, the expansion of generic drugs represents a significant decrease in resources allocated to antimicrobials, which alleviates their economic pressure.

For generic drugs to enter the market, generic medicines must perform the same level of treatment in the body as brand-name medicines. In the case of antimicrobials, the introduction of generic products is a matter of debate. Although from a theoretical point of view, both drugs should perform similarly, some experiences have questioned the efficacy of generics, compared to original antibiotics [4,5]. One study showed that the use of generic meropenem was associated with higher mortality in critically ill patients [6]. The reasons for these findings are not fully elucidated. Information on generic antimicrobials sometimes remains limited and heterogeneous, which has even caused the questioning of the production processes of these drugs [7]. This is derived from the impact related to the lack of interest of the pharmaceutical industry in the development of new antimicrobials [8,9]. However, the evidence supporting these findings remains weak due to the low quality of the available works. From a safety perspective, no studies have compared the outcomes among brand-name and generic antibiotics.

The potential impact that the introduction of a generic product has on the consumption of the rest of antibiotics is an important issue that should be highlighted. The generic entry could decrease the “control” of antimicrobial stewardship teams over its prescriptions, as these teams may be focused on more expensive drugs. As a consequence, an increase in their consumption may follow, with the risk of an augment in antimicrobial resistance [8].

Other works have assessed the impact caused by the introduction of generic antimicrobials [10,11,12]. In the last study, showing the impact of generic entry in the United States of America (USA), the number of prescriptions increased for aztreonam, cefpodoxime, ciprofloxacin, levofloxacin, and ofloxacin, while it decreased for cefdinir [10]. In Germany, the reduction in the price of fluoroquinolone was related to a 36% decline in its acquisition cost but, on the other hand, to a rise in its use of 46% [11]. Another work demonstrated that the introduction of a generic fluoroquinolone led to a rise in its consumption, which caused an increase in the *Escherichia coli* resistance rates [12]. However, these works present several limitations: they have mainly focused on the primary care setting and have not assessed the impact of a new generic antimicrobial on other members of the family or its impact on antimicrobial resistance.

The potential impact of the entry of a generic antibiotic in the market on antimicrobial consumption remains, therefore, poorly studied. The objective of this study was to analyze the modifications in the trends of antimicrobial use in an acute care hospital derived from the availability of generic formulations.

## 2. Results

During the study period, 18 drugs were included as generic antimicrobials in the hospital and were therefore analyzed (Table 1). It is important to bear in mind that the antimicrobial stewardship team was not introduced until 2011, so the present data may reflect the consumption before or after its introduction.

Before introducing the generic compound, the use of many antibiotics already showed a troubling upward trend over the years. This was notable for amoxicillin, cefixime, imipenem–cilastatin, levofloxacin, and meropenem. On the contrary, the consumption of cloxacillin and teicoplanin significantly reduced over this period.

After generic entry, the prescriptions of parenteral fosfomycin, meropenem, piperacillin/tazobactam, and vancomycin increased significantly, whereas a diminution in the use of clindamycin, imipenem–cilastatin, and levofloxacin was observed.

**Figure 1:**
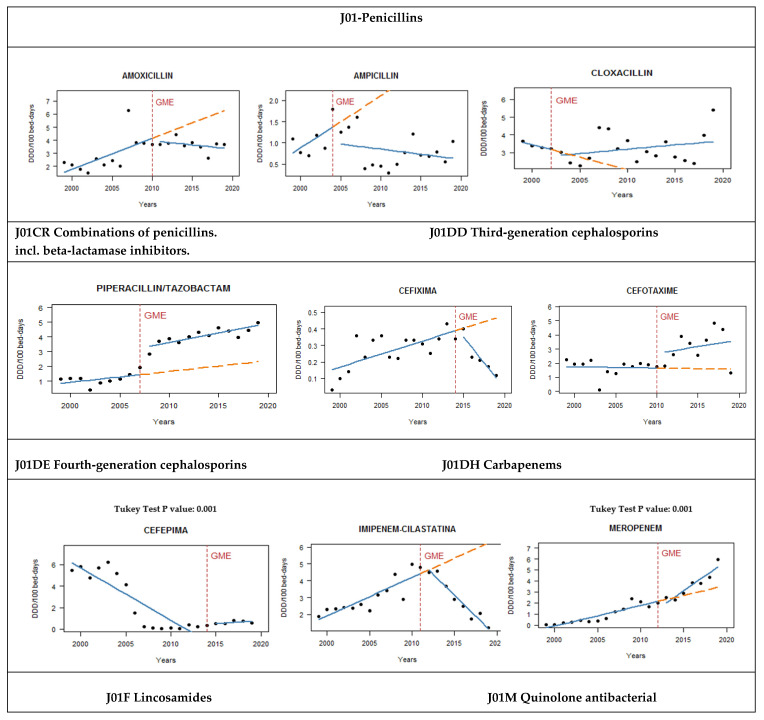

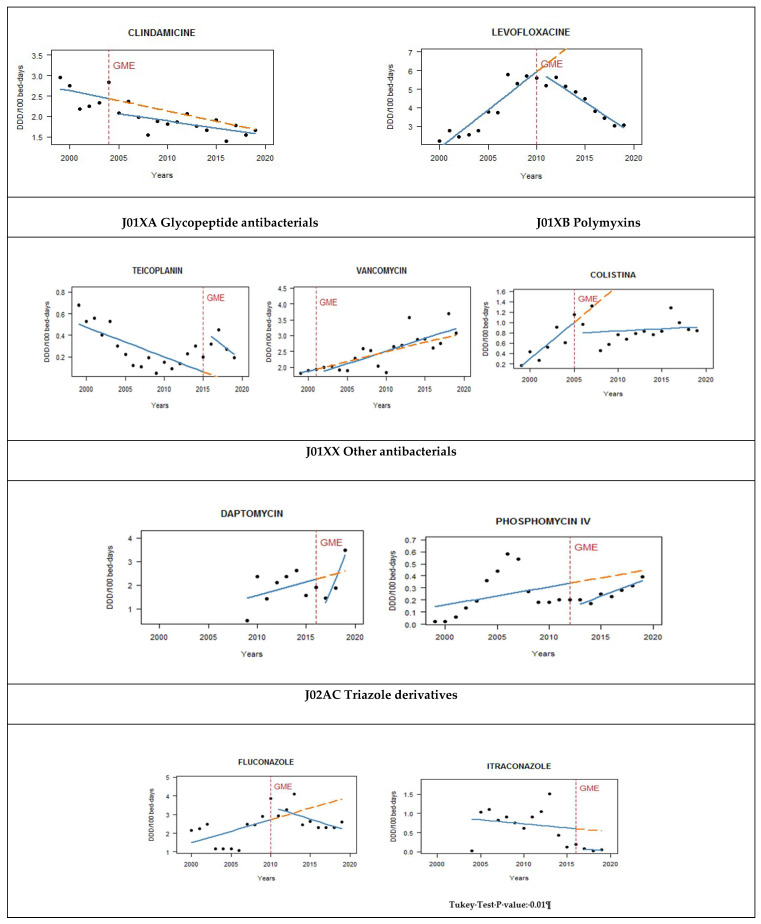
Antimicrobial consumption evolution after generic market entry: DDD—defined daily doses, GME—generic market entry.

When consumption trends before and after generic entry were compared (Table 1 and Figure 1), only cefotaxime, meropenem, and piperacillin–tazobactam showed a statistically significant upward trend. Otherwise, clindamycin and itraconazole presented a statistically significant reduction in their use.

Despite not achieving statistical significance, seven drugs (amoxicillin, ampicillin, cefixime, colistin, fluconazole, imipenem–cilastatin, and levofloxacin) presented an important reduction in antibiotic use after generic entry. On the other hand, cefepime, daptomycin, and cloxacillin showed a rise in the number of prescriptions after a downward trend before the introduction of the generic.

The findings of daptomycin, levofloxacin, and imipenem–cilastatin, although not significant, should be underlined. In the case of lipoglycopeptide, a rise in its consumption was observed after generic entry. It may be possible that statistical significance was not achieved due to the low number of years after the introduction of the generic product. The consumption of levofloxacin decreased sharply. Unfortunately, data on the consumption of ciprofloxacin were not available. Finally, as far as imipenem–cilastatin is concerned, an important downward trend was observed, even though meropenem use increased significantly.

After the introduction of the generic product, its impact on prescribers’ habits was not immediate. Overall, the effect of generic entry was observed beyond the first year post antibiotic generic entry, indicating that changes in prescription patterns take time to be observed.

## 3. Discussion

In this study, we assessed the impact of generic drug entry in the market on the prescription of antimicrobials in the hospital. When pre- and post-generic entry periods were compared, a striking and significant upward trend was found for cefotaxime, meropenem, and piperacillin–tazobactam. Itraconazole and clindamycin consumption, on the contrary, significantly decreased, compared to the previous period. The case of daptomycin, imipenem–cilastatin, and levofloxacin should also be highlighted due to their potential ecological impact, although this did not achieve statistical significance.

Although generic products represented up to two-thirds of the worldwide consumption of antimicrobial agents in 2010, the effect of generic products’ entry on antimicrobial consumption has been overall poorly described. This is concerning since generic entry may increase the number of prescriptions of these antimicrobials, which may subsequently increase bacterial resistance rates. Therefore, the knowledge of prescribing patterns after generic products’ entry is essential to establish policies that optimize antimicrobial use.

On the one hand, an alarming increase in the consumption of broad-spectrum antimicrobials of meropenem and piperacillin–tazobactam was observed. The increase in carbapenem consumption was already described in Catalonia and France [13,14]. Carbapenems were mainly used as empiric therapy for intraabdominal and urinary tract infections, the main reason for which was the suspicion of polymicrobial infection and severity [13]. In our study, two reasons may explain the increase in the use of meropenem: an increment in the incidence of multidrug-resistant organisms (data not shown) and a looser antibiotic policy at the hospital, specifically regarding empirical therapy. This last reason may be related to a reduced control by antimicrobial stewardship teams, who were unfortunately required to focus on more expensive molecules [10]. Antimicrobial stewardship teams need to face limited resources, which necessitates prioritizing those interventions that might have a greater impact [15]. Consequently, when generic products enter the market (carbapenems and others), a looser prescription policy may arise, increasing their consumption.

As regards piperacillin–tazobactam, several reasons may explain these findings. Although the consumption of carbapenems increased, a widely known strategy to reduce carbapenem consumption is the use of carbapenem-sparing agents such as piperacillin–tazobactam or amoxicillin–clavulanate [16]. Therefore, this augment may be due to a higher prescription of a carbapenem-sparing empiric agent and to an augment in its use after therapy de-escalation from carbapenems [15,17]. This fact was observed despite piperacillin–tazobactam shortage in 2016–2017. Finally, cefotaxime’s use mainly surged because of its introduction in the protocol of intraabdominal infections.

On the other hand, the use of imipenem–cilastatin, levofloxacin, and fluconazole decreased. In the case of fluoroquinolones, the reduction in their consumption may be related to the recommendations already made in old and new studies [18,19], as well as to the recommendations performed by regulatory agencies as European Medicine Agency [20]. In these documents, a restricted fluoroquinolone prescribing policy was recommended due to the potential associated adverse events. In the case of imipenem–cilastatin, the reduction in its consumption may reflect the incentive from the pharmaceutical industry in prescribing these drugs that, after patent expiration, are removed. Similar observations were also made in Germany, where a physician’s decision to prescribe an antibiotic was guided by financial incentives [21]. Another potential explanation may include a switch in prescribing patterns to meropenem, which is possibly related to the growing prevalence of nosocomial infections produced by multidrug- and extremely drug-resistant *Pseudomonas aeruginosa* infections [22]. As far as fluconazole is concerned, the decrease in its use may be due to a higher incidence of fluconazole-resistant strains such as *Candida glabrata* and a higher prescription of echinocandins [23].

The increase in the consumption of daptomycin after generic entry should be mentioned. Similar to meropenem, a looser antibiotic policy may explain these findings. A decrease in the cost of acquiring this drug would justify a decrease in the control of its prescription and, therefore, an increase in its consumption. The prevalence of infections caused by multidrug-resistant Gram-positive microorganisms such as methicillin-resistant *Staphylococcus aureus* (MRSA) remained stable during the study period [24]; therefore, it does not seem to justify this rise. The increase in the use of antibiotics against multidrug-resistant Gram-positive microorganisms has already been described in a previous work conducted in Catalonia, which found similar results [24]. A widespread and steady increase in the consumption of drugs targeting MRSA and other Gram-positive microorganisms such as daptomycin was observed among acute care hospitals, even though the incidence of MRSA remained stable.

Interestingly, the use of vancomycin did not decrease; therefore, the rise in daptomycin consumption could not be fully explained by a switch in prescribing patterns. Another potential reason for the maintenance of vancomycin consumption is the questionable behavior of daptomycin against *Enterococcus faecium* [25]. This fact has limited its use in infections caused by this microorganism, which, on the other hand, is increasingly present in hospital infections [25].

Our results are in line with those found in other works. Jensen et al. found that a significant increase in the total consumption of ciprofloxacin in ambulatory care followed the generic product’s entry in Denmark [12]. Interestingly, this finding was correlated with a higher *E. coli* resistance from urine isolates [12]. Kaier et al. also described an increasing trend of fluoroquinolone and second-generation cephalosporins use in German ambulatory care after generic entry [21]. Another study evaluated the generic launch impact in six high-income countries in the world [26]. The result of generic entry varied across countries: levofloxacin consumption significantly increased in the USA (8.6%) and the United Kingdom (UK) (4.3%), whereas declined in Germany (−9.1%). Regarding meropenem, its use declined in most of the countries except Spain, where a significant positive change in slope was found. Kallberg et al. recently assessed the effect of generic market entry on antibiotic consumption in the USA [10]. The use of aztreonam, cefpodoxime, ciprofloxacin, levofloxacin, and ofloxacin increased, whereas that of cefdinir decreased. Unfortunately, these studies presented several limitations: most of the experiences were focused on an ambulatory setting, consumption data were based on sales data, a small number of molecules were studied, and some were conducted in other countries with different healthcare systems, which hinders the potential comparisons.

It is evident that several factors play a role in the impact of generic market entry on antimicrobial consumption. The “genericization” of the antibiotics has undermined their value, as many agents that are still very effective, are very cheap, compared to their societal role [8]. When the price comes down, the product is more affordable for a larger proportion of the population, although it depends on the healthcare system [12]. On the contrary, a 10 % increase in the price would decrease consumption by 4–9%, which is mainly seen in the community rather than in the hospital [2]. The impact of antibiotic prices on patients’ adherence in the community is controversial. For some, the more the cost of a product is, the more the perceived value becomes, which may account for a higher adherence or improvement in the use. On the contrary, for others, an increase in the cost may decrease adherence, especially in settings with low economic resources [2].

Kallberg et al. described the influence of co-interventions [10]. The introduction of other (i.e., inhaled) formulations, the presence of outbreaks (i.e., anthrax), or safety reports may alter antimicrobial use [10]. The importance of vaccines introduction should also be underlined. For example, in the USA, the introduction of pneumococcal vaccines may mask a significant increase in the use of cefuroxime or even a reduction in cefdinir use after generic entry [10]. Pricing policy is also an interesting yet debatable topic that has already been discussed [2]. In our study, there were neither significant outbreaks nor vaccine policy modifications, and therefore, their influence should be limited. As previously mentioned, the safety alerts of fluoroquinolones may have reduced their consumption.

One interesting point is the impact of generic entry on the rest of the molecules of the same family. However, it is difficult to ascertain the differences between antibiotic uses for the same indications. As there are many indications for each antibiotic, it is difficult to know exactly which antibiotic would substitute another in practice. Even when the new agent has a broader spectrum of activity, its use will not entirely replace that of other agents of the same class [27]. For example, the introduction of levofloxacin in Belgium led to an overall increase in fluoroquinolone consumption [27].

The number of trade names may also play a role in the increase in antibiotic consumption after generic entry. In a European study, the number of trade names of oral antibacterial agents and community antibiotic consumption were related—more consumption was observed when more trade names were present [27].

The impact on antimicrobial consumption after generic entry was not immediate. In our study, approximately one year was needed to detect a change in antibiotic trend consumption. This is similar to other experiences and reflects that time is needed to modify prescribing patterns among providers [10,12].

One of the most important consequences of generic market entry was the troubling increase in the consumption of some antimicrobials. The driving force for the antimicrobial resistance rise is the extent of drug use [12]. This is an important issue that should be considered by antimicrobial stewardship members. Although it is true that especially new antibiotics have a great impact on the economic budget, the increase in the consumption of other cheaper drugs may also have important health and therefore economic consequences [8]. The generic entry could be considered a double-edge sword: the incorporation of generic antibiotic formulations constitutes an “oxygen balloon” in economic terms. However, antimicrobial stewardship teams should not exclude strict control of all antimicrobials from their objectives since the potential economic benefit of generic antibiotics could be diluted by an increase in bacterial resistance.

This study is not without limitations. Firstly, we were not able to include all drugs and families. Secondly, we, unfortunately, lacked microbiological data, which could have given a potential explanation for the increase in the use of some drugs.

## 4. Materials and Methods

### 4.1. Study Setting and Design

This study was conducted at the Hospital del Mar, a 400-bed tertiary hospital located in Barcelona, Spain, with an influence area of 300,000 inhabitants. The center includes two intensive care units (medical and surgical), an active program for renal transplantation, and oncology and hematology wards.

The present work was a retrospective quasi-experimental interrupted time series analysis conducted to evaluate the impact of generic market entry on antimicrobial consumption in the hospital. The evaluated outcome included antimicrobial consumption, expressed as defined daily doses (DDD)/100 bed days.

We included all the antimicrobials for systemic use for which a generic product entered the market from January 2000 to December 2019. Oral and parenteral formulations of each drug were recorded unless stated otherwise. The decision of generic entry was taken by the Pharmacy Department after reviewing the available evidence of the generic product and the fact sheet. All generic products had been approved by the Spanish Agency of Drugs. Every time a generic product was marketed, the Pharmacy Department modified purchases of the product. There was no other specific policy in relation to generic entry in the hospital. The generic entry period was defined as the first month when prescriptions of a generic product were first recorded [10]. Afterward, we selected all the drugs in which there were enough data to perform statistical analysis. These drugs included oral amoxicillin, parenteral ampicillin, parenteral cefepime, oral cefixime, parenteral cefotaxime, oral and parenteral clindamycin, parenteral cloxacillin, parenteral and nebulized colistin, parenteral daptomycin, oral and parenteral fluconazole, parenteral fosfomycin, parenteral imipenem-cilastatin, oral itraconazole, oral and parenteral levofloxacin, parenteral meropenem, parenteral piperacillin–tazobactam, parenteral teicoplanin, and parenteral vancomycin.

Our hospital implemented the antimicrobial stewardship program from 1 January 2011 to 31 March 2011. This team includes specialists in infectious diseases, pharmacy, microbiology, and intensive care medicine [28]. Among the different interventions, a computer application for the prescription of antimicrobials was developed within patients’ electronic medical records. When one of the selected antimicrobials was prescribed, providers needed to justify the indication and include the duration of the treatment (with automatic discontinuation on the day set). Thereafter, information on the cost of the treatment was displayed on the screen. The antimicrobials included were linezolid, aztreonam, echinocandins (anidulafungin, caspofungin, and micafungin), carbapenems (ertapenem, imipenem, and meropenem), daptomycin, tigecycline, voriconazole, and liposomal amphotericin B [29]. These antimicrobials were monitored even after generic entry. A member of the working group reviewed and reassessed these indications during the first 24 to 72 h and thereafter daily.

### 4.2. Data Collection

The consumption data were obtained by the Pharmacy Department through a computer tool on a monthly basis from January 2000 to December 2020. However, to avoid the inferences of the COVID-19 pandemic, information until December 2019 was only included. Consumption data were based on dispensation, rather than administration, data. We acknowledge the potential limitations of this methodology since the consumption may be overestimated and may not reflect which drug the patient has finally received.

All antibiotic consumption data were obtained from the Anatomical Therapeutic Chemical/Defined Daily Dose (ATC/DDD) classification system. This methodology was developed by the WHO Collaborating Center for Drug Statistics Methodology [30]. The ATC/DDD system was used to monitor adult hospital anti-infectives for systemic consumption. The admission and discharge days were considered as a single day [31]. Adult antibacterial and antimycotic consumption was calculated on a yearly basis and expressed as DDD/100 bed days.

### 4.3. Statistical Analysis

Differences in specific antibacterial consumption before and after generic market entry were visually analyzed using a simple linear model to detect possible significant trend changes. Corresponding confidence intervals (CIs) and p-values for each antibacterial trend were computed for pre-generic and post-generic entries separately.

To find significant variation between pre-GME and post-GME, an interrupted time series analysis (ITSA) was implemented, and Tukey’s *p*-value was obtained.

For all statistical analyses, 95% CIs were calculated. *p*-values of <0.05 were considered statistically significant. A bilateral distribution was assumed for all p-values. The analyses were performed using R v4.0.4 statistical software (R foundation for Statistical Computing, Vienna, Austria).

## 5. Conclusions

Generic entry of drugs such as meropenem or piperacillin–tazobactam led to an increase in the consumption of these broad-spectrum agents, whereas in the case of clindamycin, consumption was reduced. Antimicrobial stewardship should maintain, despite the lower associated costs of generic products, the monitoring of the prescriptions of these agents to try to reduce their potential ecological consequences.

## Figures and Tables

**Table 1:** Antimicrobial consumption trends before and after generic entry.

	Pre-Generic Trend			Post-Generic Trend			
	Est. Coefficient	95% CI	*p*-Value	Est. Coefficient	95% CI	*p*-Value	Tukey’s Test *p*-Value
J01 Antibacterials for systemic use							
**J01C Penicillins**							
*Cloxacillin*	−0.14	[−0.28, −0.002]	0.049	0.05	[−0.043, 0.14]	0.277	0.92
*Amoxicillin*	0.24	[0.033, 0.438]	0.027	−0.07	[−0.205, 0.074]	0.302	0.15
*Ampicillin*	0.12	[−0.121, 0.365]	0.235	−0.02	[−0.075, 0.027]	0.333	0.24
**J01CR Combinations of penicillins. incl. beta-lactamase inhibitors**							
***Piperacillin–tazobactam***	**0.07**	**[**−**0.04, 0.186]**	**0.17**	**0.13**	**[0.068, 0.186]**	**<0.001**	**<0.001**
**J01DBCDE Cephalosporins**							
J01DD Third-generation cephalosporins							
*Cefixime*	0.02	[0.006, 0.025]	0.003	−0.06	[−0.109, −0.015]	0.024	0.22
***Cefotaxime***	−**0.01**	**[**−**0.121, 0.104]**	**0.865**	**0.09**	**[**−**0.282, 0.466]**	**0.58**	**<0.001**
J01DE Fourth-generation cephalosporins							
*Cefepime*	−0.48	[−0.632, −0.337]	<0.001	0.004	[−0.106, 0.186]	0.447	0.17
**J01DH Carbapenems**							
*Imipenem–cilastatina*	0.23	[0.138, 0.319]	<0.001	−0.50	[−0.621, −0.374]	<0.001	0.62
***Meropenem***	**0.18**	**[0.133, 0.236]**	**<0.001**	**0.54**	**[0.308, 0.782]**	**0.002**	**<0.001**
**J01F Lincosamides**							
***Clindamycin***	−**0.05**	**[**−**0.288, 0.189]**	**0.594**	−**0.03**	**[**−**0.06,** −**0.01]**	**0.011**	**<0.001**
**J01M Quinolone antibacterials**							
*Levofloxacine*	0.41	[0.285, 0.533]	<0.001	−0.35	[−0.433, −0.263]	<0.001	0.66
**J01X Other antibacterials** J01XA Glycopeptide antibacterials							
*Vancomycin*	0.06	[−0.087, 0.207]	0.121	0.08	[0.045–0.115]	<0.001	0.13
*Teicoplanin* J01XB Polymyxins	−0.03	[−0.042, −0.013]	<0.001	−0.06	[−0.247, 0.133]	0.325	0.58
*Colistin* J01XX Other antibacterials	0.14	[0.051, 0.23]	0.01	0.01	[−0.027, 0.044]	0.609	0.12
Daptomycin	0.11	[−0.137, 0.365]	0.309	1.02	[−3.277, 5.307]	0.205	0.64
Intravenous fosfomycin	0.02	[−0.01, 0.04]	0.218	0.03	[0.017, 0.047]	0.003	0.94
**J02A Antimycotics for systemic use**							
J02AC Triazole derivatives							
*Fluconazole*	0.12	[−0.055, 0.303]	0.152	−0.13	[−0.287, 0.02]	0.079	0.09
***Itraconazole***	−**0.02**	**[**−**0.094, 0.052]**	**0.537**	−**0.02**	**[**−**0.345, 0.315]**	**0.667**	**0.01**

## Data Availability

The data presented in this study are available on request from the corresponding author.
